# Mesh placement and risk of reoperation for recurrence after incisional hernia repair: a nationwide register-based cohort study

**DOI:** 10.1007/s10029-026-03690-y

**Published:** 2026-05-25

**Authors:** Camilla Witthøft, Usamah Ahmed, Jacob Rosenberg, Jason Joe Baker

**Affiliations:** Center for Perioperative Optimization, Department of Surgery, Copenhagen University Hospital - Herlev and Gentofte, Borgmester Ib Juuls Vej 1, 2730 Herlev, Denmark

**Keywords:** Incisional hernia, Ventral hernia, Reoperation, Recurrence, Surgical technique

## Abstract

**Purpose:**

Retromuscular mesh placement is recommended for incisional hernia repair, but the current certainty of evidence remains low. This study aimed to compare the risk of reoperation for recurrence among adults undergoing incisional hernia repair with onlay, retromuscular, preperitoneal, and intraperitoneal onlay mesh (IPOM).

**Methods:**

This study used prospectively collected data from the Danish Ventral Hernia Database that were linked to the Danish National Patient Register and the Danish Civil Registration System. We included patients undergoing elective incisional hernia repair with defect widths  ≤ 10 cm operated between 2007 and 2025. The primary outcome was reoperation for recurrence, analyzed using Cox regression, and included subgroup analyses of defect width, surgical approach, and type of previous incision.

**Results:**

In total, 5,375 patients were included, of whom 14% received a preperitoneal mesh placement, 22% retromuscular placement, 30% IPOM with defect closure, and 34% onlay placement. Compared with preperitoneal placement, onlay was associated with a higher risk of reoperation (HR 2.62, 95% CI 1.73–3.95; p < 0.001). Onlay was associated with a higher risk than all other placements. However, this association was not observed in subgroup analyses for defect widths  ≤ 2 cm (p = 0.058), in which retromuscular placement was associated with a significantly increased risk of reoperation.

**Conclusion:**

Onlay mesh placement for defect widths  > 2 cm and retromuscular mesh placement for defect widths  ≤ 2 cm were associated with higher risk of reoperation for recurrence.

**Supplementary Information:**

The online version contains supplementary material available at 10.1007/s10029-026-03690-y.

## Introduction

Incisional hernias constitute a heterogeneous group of hernias that vary considerably in size, anatomical location, and clinical presentation [1]. Current guidelines from the European Hernia Society recommend retromuscular mesh placement for incisional hernia repair [2]. However, this recommendation is supported by very low-certainty evidence, largely due to statistical heterogeneity, publication bias, and clinical heterogeneity, including variations in surgical techniques and patient populations [2]. Moreover, several previous studies have pooled primary ventral and incisional hernias, despite recommendations to analyze these entities separately due to differences in patient characteristics and clinical outcomes [3, 4]. In addition to retromuscular placement, onlay, preperitoneal, and intraperitoneal onlay mesh (IPOM) techniques are widely used in clinical practice [5]. Nevertheless, the optimal mesh position regarding recurrence and complications remains debated [6]. A systematic review [7] comparing retromuscular with onlay mesh placement reported moderate level of heterogeneity, potential risk of bias, and a limited number of high-quality studies. Although recent studies [8, 9] suggest a trend toward retromuscular and preperitoneal placements, the existing evidence remains inconclusive. Reported recurrence rates also vary widely, with one study indicating that more than half of patients experience recurrence after incisional hernia repair [10]. Taken together, these limitations highlight the need for large-scale studies focusing specifically on incisional hernias.

Therefore, the aim of this nationwide register-based cohort study was to compare the risk of reoperation for recurrence among adults undergoing incisional hernia repair with onlay, retromuscular, preperitoneal, and intraperitoneal mesh placement.

## Methods

This nationwide register-based cohort study used prospectively collected data from the Danish Ventral Hernia Database [11] and linked these data to the Danish Civil Registration System [12] and the Danish National Patient Register [13]. This study was conducted in accordance with the Reporting of studies Conducted using Observational Routinely-collected health Data (RECORD) guideline [14].

Surgeons from public and private hospitals, as well as private clinics, are required to register data on patient characteristics and operative details following ventral hernia repair into the Danish Ventral Hernia Database [11]. In 2024, the nationwide registration rate was 82% [15]. Validation against medical records has demonstrated an accuracy of 89–99% [16]. To enable complete follow-up and linkage across national registers, the Danish Civil Registration System is used, as all Danish citizens are assigned a unique personal identification number [12], which provides information on vital status, including death or emigration, along with the corresponding dates. Using this identifier, all contacts between Danish residents and the healthcare system are recorded in the Danish National Patient Registry using this identification number [13]. Together, these registers enable complete follow-up of the included patients. Data were extracted for all patients registered from January 2007, when the Danish Ventral Hernia Database was fully established, until October 29, 2025, the date of data extraction. In addition, 59 operations registered before 2007 as part of a pilot test of the database were also included in the study.

We included adults undergoing elective incisional hernia repair with mesh placed in an onlay, retromuscular, preperitoneal, or IPOM position. Only patients with an index repair registered in the Danish Ventral Hernia Database were included, as registration was required to obtain information on mesh placement. Physiomesh™ (Ethicon) was excluded because it has been withdrawn from the market [17] due to high failure rates [18]. We excluded procedures involving component separation, contaminated fields, non-mesh repair, repairs where the first recorded operation was for a recurrent hernia, and repairs using other mesh placements. IPOM without defect closure was also excluded because it carries a higher risk of recurrence than IPOM with defect closure (IPOM +) [19]. Patients were excluded if the defect width was  > 10 cm or registered as 0 cm. Finally, patients who had emigrated from Denmark were excluded, as subsequent hernia recurrence could not be reliably ascertained.

Reoperation was used as a proxy for recurrence. It was defined as surgery for recurrent incisional hernia following the index repair. Duplicates were identified using the personal identification number, date of operation, and hernia type, and were subsequently removed. When a reoperation was registered only in the Danish National Patient Register, available data were limited to the personal identification number, procedure code, and procedure date [13]. Based on the procedure codes, the hernia type could be determined, thereby allowing identification of reoperations. Procedure codes follow the Nordic Medico-Statistical Committee classification, with an added “K” prefix, and incisional hernias are coded as “KJAD” [20]. Potential confounders were identified and extracted for analysis, including sex, age [21], hernia defect width [4], and Charlson Comorbidity Index (a weighted score reflecting comorbidity) [22]. For analytical purposes, patients with a Charlson Comorbidity Index  ≥ 4 were merged into a single category due to small patient numbers. Defect width was categorized as  ≤ 2 cm,  > 2–6 cm, and  > 6–10 cm, in accordance with the Danish Ventral Hernia Database guidelines [23]. Follow-up was defined as the time from the index operation to the first reoperation, death, or the date of data extraction. For subgroup analyses, incisional hernias were further classified as midline or non-midline. Midline hernias were defined according to the European Hernia Society (EHS) classification (M1–M5), including epigastric, umbilical, infraumbilical, supraumbilical, and subxiphoid locations [24]. As this variable was introduced into the Danish Ventral Hernia Database in 2020, information on hernia location was not available for procedures registered before that year. However, another variable classified the incisional hernia with a vertical or transverse incision, and if the hernia location was not recorded according to the EHS classification, the hernia was categorized as midline if it was recorded with a vertical incision. If not, the hernia was classified as non-midline. The study size was determined by including all eligible patients registered in the database during the study period.

Categorical variables were presented as crude rates and percentages. Continuous variables were assessed for normality using histograms and Q–Q plots. As most continuous variables were not normally distributed, they were all presented as medians with interquartile ranges. The unadjusted cumulative risk of reoperation was illustrated with a Kaplan–Meier plot, and differences were assessed with the log-rank test. To compare time to reoperation across all combinations of the four mesh placements, pairwise log-rank tests were performed, and Holm correction was applied to adjust for multiple comparisons [25]. To further evaluate the risk of reoperation, Cox regression was performed, adjusting for sex, age, defect width, and Charlson Comorbidity Index. Separate models were fitted using each mesh placement as the reference category, and the placement associated with the lowest recurrence risk was selected as the primary reference in all analyses. A two-sided p-value < 0.05 was considered statistically significant. Prespecified subgroup analyses were conducted according to defect width groups (≤ 2,  > 2–6, and  > 6–10 cm), surgical approaches (open, laparoscopic, and robotic approach), body mass index (BMI) grouped  ≤ 30 and  > 30 kg/m^2^, and hernia location (midline and non-midline). Furthermore, retromuscular and preperitoneal mesh placements were analyzed together as sublay in another analysis. A sensitivity analysis was performed, excluding patients who received preoperative Botulinum toxin (Botox®). As Botulinum toxin was introduced into the Danish Ventral Hernia Database in 2017, patients operated before 2017 were included in this analysis. Statistical analyses were performed using RStudio, version 4.5.0 [26].

This study was approved by the Danish Healthcare Quality Institute and the Danish Data Protection Agency (journal no.: p-2025–18750). According to Danish legislation, neither ethics approval nor patient consent is required for studies based on registry data [27].

## Results

**Fig. 1:**
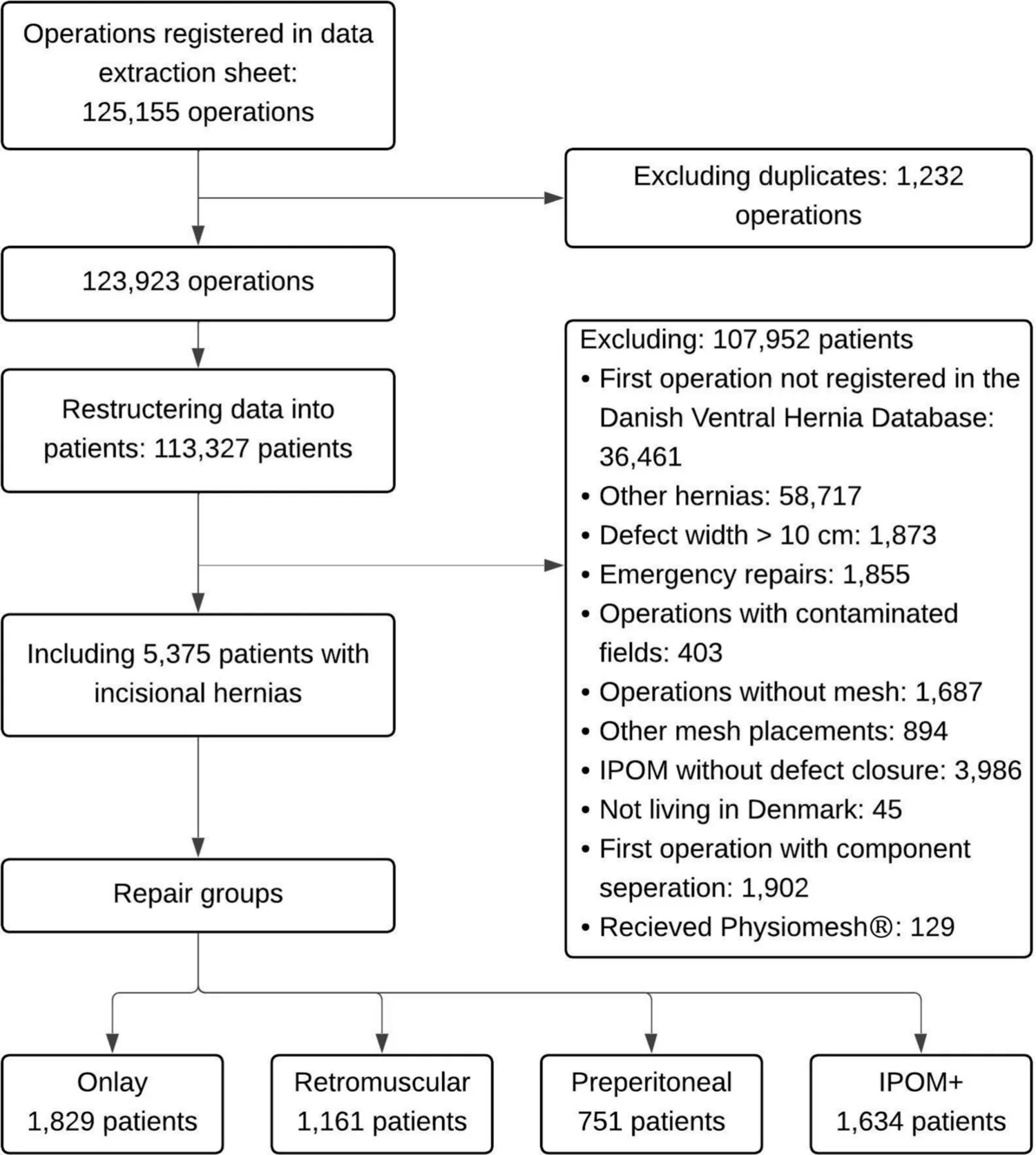
Flowchart of data selection and cleaning

A total of 5,375 patients were included in the study (Fig. 1). Preperitoneal mesh placement accounted for 14% of the repairs, retromuscular for 22%, IPOM + for 30%, and onlay for 34%. Notably, approximately two-thirds of patients in the preperitoneal group were female, whereas sex distribution was more balanced in the other mesh placement groups. The median follow-up was five years for onlay, retromuscular, and preperitoneal repairs, compared with eight years for IPOM +. Retromuscular repair was more frequently used for larger defects, with 30% of retromuscular repairs performed in hernias measuring  > 6–10 cm. Onlay repairs were performed using an open approach, retromuscular repairs were predominantly open, IPOM +  was mainly performed laparoscopically, and preperitoneal mesh placement showed an even distribution between open and laparoscopic approaches (Table 1).

**Table 1:** Patient characteristics. Values are n (%) unless otherwise indicated. IPOM + : intraperitoneal mesh placement with defect closure; n: number of patients; IQR: interquartile range

		Onlay n = 1,829	Retromuscular n = 1,161	Preperitoneal n = 751	IPOM + n = 1,634
Age (years), median (IQR)		60 (48–69)	63 (52–71)	58 (48–68)	61 (51–70)
Female sex		1,004 (55)	568 (49)	465 (62)	916 (56)
BMI, kg/m^2^, median (IQR)		28 (25–31)	28 (25–32)	28 (25–31)	28 (26–32)
Missing data		767 (42)	266 (23)	210 (28)	897 (55)
Hernia defect width, cm					
≤ 2 cm		988 (54)	105 (9)	434 (58)	391 (24)
> 2–6 cm		669 (37)	704 (61)	287 (38)	1,053 (64)
> 6–10 cm		172 (9)	352 (30)	30 (4)	190 (12)
Surgical approach					
Open		1,829 (100)	946 (81)	345 (46)	294 (18)
Laparoscopic		0 (0)	54 (5)	308 (41)	1,320 (81)
Robot		0 (0)	161 (14)	98 (13)	20 (1)
Length of hospital stay, days, median (IQR)		0 (0–1)	3 (0–5)	0 (0–0)	1 (0–2)
Charlson Comorbidity Index					
0		868 (47)	444 (38)	449 (60)	676 (41)
1		311 (17)	162 (14)	104 (14)	278 (17)
2		363 (20)	282 (24)	136 (18)	370 (23)
3		136 (7)	114 (10)	30 (4)	143 (9)
≥ 4		151 (8)	159 (14)	32 (4)	167 (10)
Follow-up (years), median (IQR)		5 (3–9)	5 (3–7)	5 (3–8)	8 (5–10)
Reoperation		191 (10)	63 (5)	26 (3)	93 (6)

**Fig. 2:**
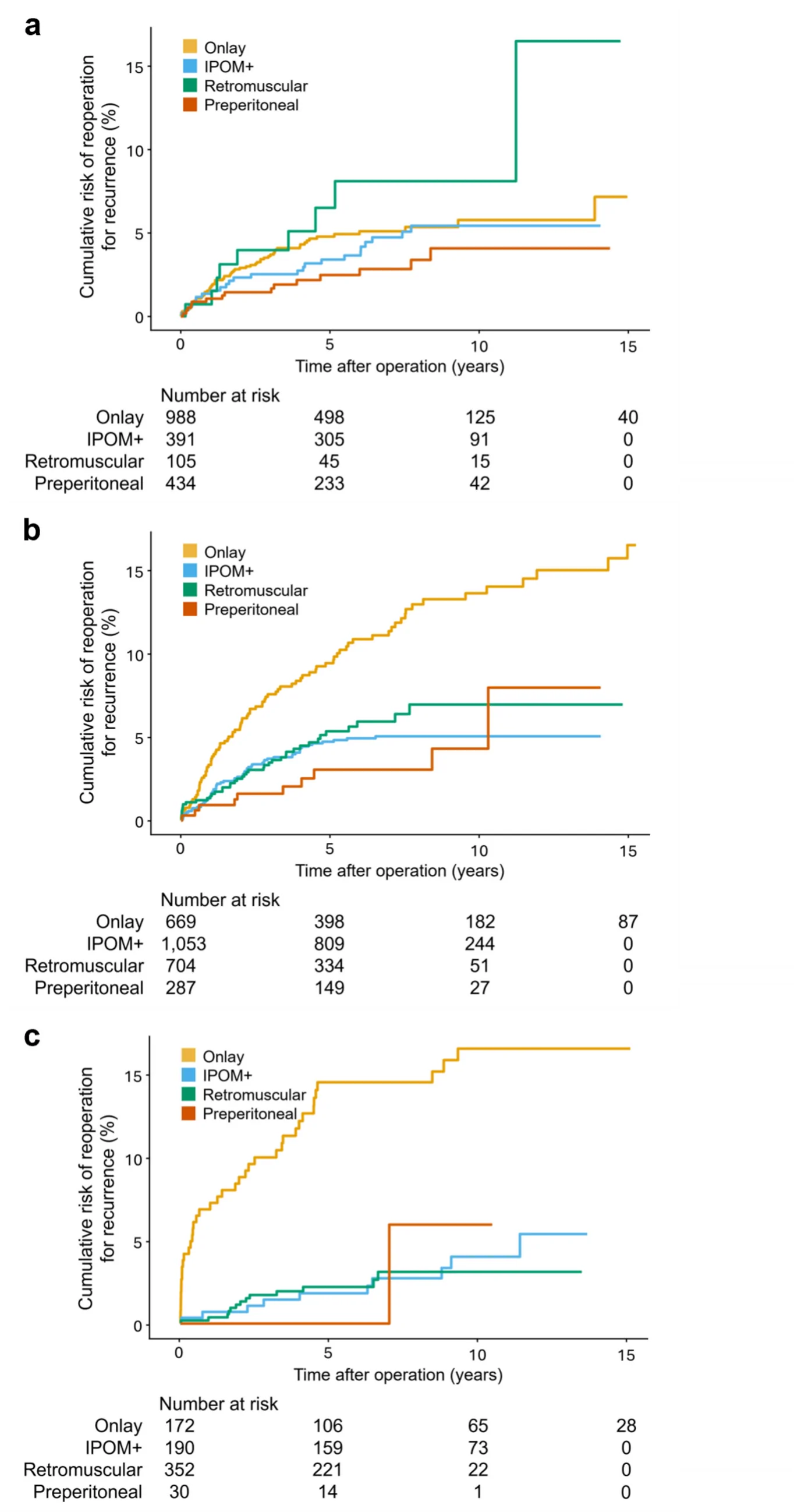
Kaplan–Meier plots showing reoperation rates stratified by mesh placements. Patients censored at death or time of data extraction. a: defect widths  ≤ 2 c; b:  > 2–6 cm; c: > 6–10 cm

**Fig. 3:**
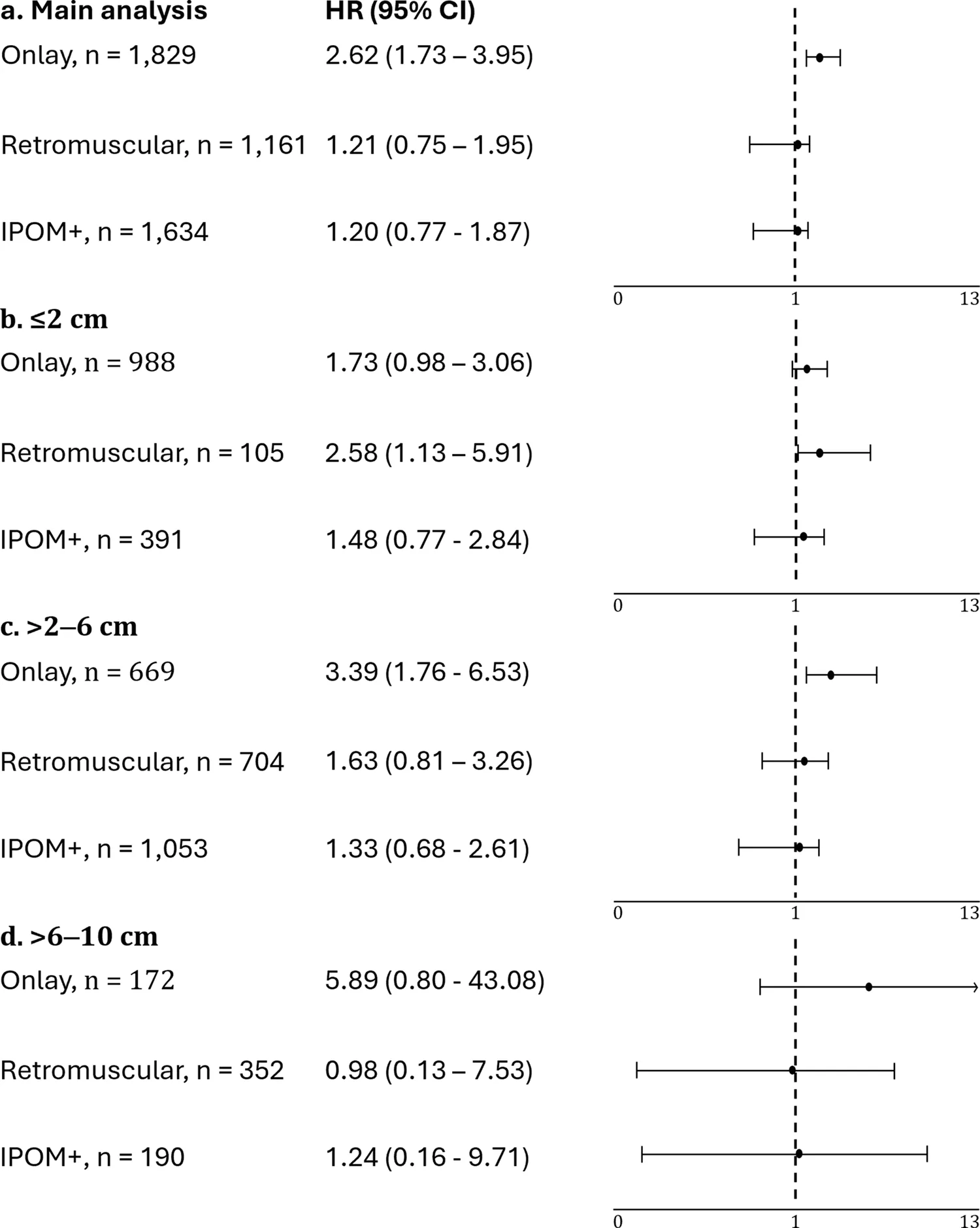
Forest plots illustrating results from Cox regression. Forest plots for the main analysis and subgroup analyses for defect widths  ≤ 2 cm,  > 2–6 cm, and  > 6–10 cm. The vertical line represents preperitoneal mesh placement as reference at hazard ratio = 1. The x-axis shows hazard ratios, where values above 1 indicate increased risk and values below 1 indicate decreased risk compared with preperitoneal mesh placement. The square represents the estimate of the hazard ratio, and the error bars indicate 95% confidence intervals. HR: hazard ratio; CI: confidence intervals

Onlay repair was associated with the highest crude reoperation rate (10%) compared with the other mesh placements (Table 1). Kaplan–Meier plots demonstrating cumulative reoperation rates over time showed that patients undergoing onlay repair had the highest risk of reoperation (Fig. 2). For defect widths >2 cm, retromuscular repair and IPOM +  showed comparable reoperation rates during the first five years of follow-up, whereas preperitoneal mesh placement was associated with the lowest reoperation rate during this period. Pairwise log-rank testing showed that onlay repair was associated with a significantly higher risk of reoperation compared with all other mesh placements (p < 0.001). In the adjusted Cox regression analysis, onlay mesh placement was associated with an increased risk of reoperation compared with preperitoneal placement (hazard ratio (HR) 2.62, 95% confidence interval (CI) 1.73–3.95; p < 0.001) (Table 2 and Fig. 3a). No significant differences were observed for retromuscular or IPOM +  repair compared with preperitoneal placement. Additional Cox regression models using alternative reference groups (onlay, retromuscular, and IPOM +) consistently showed that onlay repair was associated with a higher risk of reoperation than all other mesh placements (Supplementary Information S1). In subgroup analyses stratified by defect width, retromuscular mesh placement was associated with a higher risk of reoperation than preperitoneal placement for defects  ≤ 2 cm (Fig. 3b-d and Supplementary Information S2). In this subgroup, onlay repair was not associated with an increased risk compared with preperitoneal mesh placement (HR 1.73, 95% CI 0.98–3.06; p = 0.058). For defects  > 2–6 cm, onlay repair was associated with a higher risk of reoperation compared with preperitoneal placement (HR 3.39, 95% CI 1.76–6.53; p < 0.001). For defects  > 6–10 cm, no statistically significant differences were observed between mesh placements; however, the HR followed the same pattern as in the main analysis (Fig. 3b–d). In open procedures, onlay repair was associated with an increased risk of reoperation compared with preperitoneal placement (HR 2.73, 95% CI 1.52–4.91; p < 0.001). No significant differences were observed in laparoscopic or robotic approaches (Supplementary Information S3). For BMI ≤ 30 kg/m^2^, all mesh placements were associated with an increased risk of reoperation compared with preperitoneal mesh placement, whereas no significant differences were observed for BMI  > 30 kg/m^2^, and onlay repair was not associated with an increased risk (Supplementary Information S4). Onlay repair was associated with an increased risk of reoperation compared with preperitoneal placement in both midline (HR 2.92, 95% CI 1.18–7.23; p = 0.021) and non-midline incisional hernias (HR 2.42, 95% CI 1.51–3.89; p < 0.001) (Supplementary Information S5). Analyzing retromuscular and preperitoneal mesh placements together were consistent with the main analysis (Supplementary Information S6). Excluding patients treated with Botulinum toxin (Botox®) (n = 111), did not change the main findings.

**Table 2:** Main analysis. HR: hazard ratio; CI: confidence intervals; IPOM + : intraperitoneal mesh placement with defect closure

Characteristics		HR (95% CI)	P value
Female sex		0.88 (0.72–1.09)	0.239
Age, per year		0.99 (0.98–1.00)	0.026
Defect width, per cm		1.08 (1.03–1.12)	< 0.001
Charlson Comorbidity Index			
0		Reference	─
1		1.19 (0.88–1.59)	0.255
2		1.15 (0.86–1.53)	0.346
3		1.57 (1.07–2.29)	0.020
≥ 4		1.10 (0.74–1.64)	0.625
Mesh placement			
Preperitoneal		Reference	─
Onlay		2.62 (1.73–3.95)	< 0.001
Retromuscular		1.21 (0.75–1.95)	0.426
IPOM +		1.20 (0.77–1.87)	0.416

## Discussion

This nationwide, register-based cohort study investigated the association between mesh placement and the risk of reoperation for recurrence. Overall, onlay mesh placement was associated with a higher risk of reoperation for recurrence compared with retromuscular, preperitoneal, and IPOM +. However, retromuscular mesh placement was associated with an increased risk of reoperation in defects  ≤ 2 cm, whereas onlay was not. For BMI  ≤ 30 kg/m^2^ preperitoneal mesh decreased the risk of reoperation compared to all other placements. For defect widths  > 6–10 cm and in patients with BMI  > 30 kg/m^2^, no significant differences in reoperation risk were observed between mesh placements.

The observed differences between mesh placements may be partly explained by underlying pathophysiological mechanisms. In our cohort, all onlay repairs were performed using an open approach, which has been associated with a higher risk of wound complications compared with laparoscopic approach [28]. Surgical site infection may impair mesh integration and have been identified as risk factor for recurrence [29]. The existing literature suggests potential advantages of retromuscular and preperitoneal mesh placement compared with onlay; however, firm conclusions remain difficult to draw. Previous studies, including one randomized controlled trial [30] and one cohort study [31], both reported superior outcomes for retromuscular and preperitoneal mesh placements compared with onlay, yet both were limited by smaller sample sizes and with less extensive follow-up compared with our study. In contrast, another randomized controlled trial [32] comparing onlay and retromuscular mesh placement found no difference in recurrence rates. That study included only midline incisional hernias and excluded patients with severe pulmonary and cardiac diseases, resulting in a more homogeneous population, but had a limited sample size (n = 100). Similarly, in a cohort [33] comparing retromuscular with IPOM using pairwise propensity score matching, no difference in recurrence was observed; however, follow-up was limited to 12 months. Taken together, the conflicting evidence may be explained by differences in patient selection, sample size, adjustment for confounders, and duration of follow-up.

This study has several strengths. It is a large, nationwide cohort study including data from both private and public hospitals, providing high external validity. The risk of recall bias was small, as data were prospectively collected, and selection bias was limited because registration in the database is mandatory. The study was further strengthened by adjustment for relevant confounders [4, 21, 22]. In addition, nationwide coverage ensures complete capture of reoperations, including those performed at hospitals other than the index institution [34]. Finally, the publicly funded Danish healthcare system mitigates the impact of socioeconomic factors, including income and insurance status, on study outcomes. However, some limitations should also be acknowledged. First, using reoperation as a proxy for recurrence underestimates the true recurrence rate [35]; however, we assume that the proportion of reoperations for recurrence and true recurrence are comparable across mesh placement groups. Second, as with all registry-based studies, there is a risk of misclassification, including hernia type, location, definition of reoperation, and linkage between index repair and reoperation; however, the database has demonstrated high accuracy (89–99%) in a previous validation study [16]. Furthermore, for a subset of patients with missing information on hernia location, vertical incision type was used as a proxy for midline hernia, although some misclassification cannot be excluded. Third, the absence of patient-reported outcomes such as pain or quality of life [36] is a limitation, as reoperation may not reflect patient satisfaction. Fourth, some subgroup analyses with small sample sizes (defect width  > 6–10 cm, robotic approach, and BMI  > 30 kg/m^2^) increase the risk of type II error; these findings should therefore be interpreted with caution. Although we adjusted for several confounders, we did not adjust for surgeon volume or experience, smoking, BMI, diabetes, or chronic lung diseases, all of which may have influenced the risk of recurrence [21]. BMI and smoking were introduced into the Danish Ventral Hernia Database in 2017 and were therefore not included in the main analysis due to insufficient data coverage. These factors may also influence the choice of mesh placement [8]. However, we performed a subgroup analysis among patients with available BMI data. The subgroup analysis for defect widths  ≤ 2 cm showing that retromuscular placement increased the risk of reoperation should be interpreted with caution, as the subgroup included only 105 participants with retromuscular mesh placement, increasing the risk of type I error. The long inclusion period may have introduced temporal variation in surgical techniques and materials, which could have influenced outcomes [6, 37]. In addition, short-term postoperative outcomes were not included, as the primary focus of this study was on long-term outcomes, and the available data did not capture all postoperative complications. Finally, we did not adjust for mesh fixation; one study [38] has shown that suture fixation may reduce recurrence in IPOM repair. Mesh material was neither included in the analysis, which could also influence recurrence [17].

This nationwide, register-based cohort study provides evidence of an association between mesh placement and the risk of recurrence. Overall, the findings support a shift away from onlay mesh placement for incisional hernias with defect widths  > 2 cm [6], given its association with a higher risk of reoperation for recurrence. Furthermore, IPOM has been associated with potentially adverse outcomes [9], and mesh placed in the retromuscular or preperitoneal positions may therefore be preferred for hernias  > 2 cm. In addition to mesh placement, other aspects of incisional hernia repair should be considered, including fixation [38], surgeons’ experience and volume [39], and mesh materials, as these have shifted substantially over time [37]. Furthermore, suture material used in defect closure may also influence outcomes. Although one study found no difference between suture materials for primary ventral hernia repair [40], it remains unclear whether these findings are applicable to incisional hernias. Beyond recurrence, patient-reported outcomes are of major importance in hernia repair, including chronic pain, foreign body sensation, and quality of life [36]. These outcomes are imperative to investigate, as incisional hernia is a benign condition, and patients undergo surgery to improve their quality of life [36].

## Conclusions

Onlay mesh placement was associated with a higher risk of reoperation compared with all other mesh placements in incisional hernia repair, although not reaching statistical significance in subgroups for defect widths  > 6–10 cm and BMI  > 30 kg/m^2^. For defect widths  ≤ 2 cm, retromuscular mesh placement may be associated with a higher risk of reoperation for recurrence, however, the sample size was too small to draw a firm conclusion.

## Supplementary Information

Below is the link to the electronic supplementary material.

Supplementary file1 (DOCX 46 KB)

## Data Availability

Due to Danish law, data are not available.
