# Supplementary material for: Mesh placement and risk of reoperation for recurrence after incisional hernia repair: a nationwide register-based cohort study
Source: Hernia. 2026 May 25;30(1):228. doi: 10.1007/s10029-026-03690-y (PMC13201287; doi:10.1007/s10029-026-03690-y)
Supplement: Supplementary file 1 — Supplementary file1 (DOCX 46 KB) [file 10029_2026_3690_MOESM1_ESM.docx]

**Title:** Mesh placement and risk of reoperation for recurrence after incisional hernia repair: a nationwide register-based cohort study

**Journal:** Hernia

**Authors:** Camilla Witthøft, Usamah Ahmed, Jacob Rosenberg, Jason Joe Baker

Center for Perioperative Optimization, Department of Surgery, Copenhagen University Hospital - Herlev and Gentofte, Borgmester Ib Juuls Vej 1, DK-2730 Herlev, Denmark

**Corresponding author:** Camilla Witthøft, e-mail: [camillawitthoft@outlook.dk](mailto:camillawitthoft@outlook.dk)

**Table S1** Variations of the main analysis with other references than preperitoneal

|  | | HR (95% CI) | P value |
| --- | --- | --- | --- |
| Mesh placement | |  |  |
|  | Onlay | Reference | ─ |
|  | Retromuscular | 0.46 (0.34 – 0.63) | <0.001 |
|  | Preperitoneal | 0.38 (0.25 – 0.58) | <0.001 |
|  | IPOM+ | 0.46 (0.36 – 0.59) | <0.001 |
| Mesh placement | |  |  |
|  | Retromuscular | Reference | ─ |
|  | Onlay | 2.16 (1.59 – 2.92) | <0.001 |
|  | Preperitoneal | 0.82 (0.51 – 1.33) | 0.426 |
|  | IPOM+ | 0.99 (0.71 – 1.37) | 0.951 |
| Mesh placement | |  |  |
|  | IPOM+ | Reference | ─ |
|  | Onlay | 2.18 (1.69 – 2.80) | <0.001 |
|  | Retromuscular | 1.01 (0.73 – 1.40) | 0.951 |
|  | Preperitoneal | 0.83 (0.54 – 1.29) | 0.416 |

Footnotes: Variations of the main analysis with other references than preperitoneal. Adjusted for sex, age, defect width, and Charlson Comorbidity Index. HR: hazard ratio; IPOM+: intraperitoneal mesh placement with defect closure.

**Table S2** Subgroup analyses for defect width

|  | | HR (95% CI) | P value |
| --- | --- | --- | --- |
| Defect width ≤2 cm, n = 1,918 | |  |  |
| Female sex | | 1.14 (0.78 – 1.67) | 0.501 |
| Age, per year | | 1.00 (0.98 – 1.01) | 0.551 |
| Charlson Comorbidity Index | |  |  |
|  | 0 | Reference | ─ |
|  | 1 | 0.88 (0.48 – 1.59) | 0.667 |
|  | 2 | 1.74 (1.06 – 2.85) | 0.029 |
|  | 3 | 2.06 (1.01 – 4.19) | 0.046 |
|  | ≥4 | 0.76 (0.27 – 2.14) | 0.603 |
| Mesh placement | |  |  |
|  | Preperitoneal | Reference | ─ |
|  | Onlay | 1.73 (0.98 – 3.06) | 0.058 |
|  | Retromuscular | 2.58 (1.13 – 5.91) | 0.025 |
|  | IPOM+ | 1.48 (0.77 – 2.84) | 0.235 |
| Defect width >2–6 cm, n = 2,713 | |  |  |
| Female sex | | 0.71 (0.53 – 0.94) | 0.017 |
| Age, per year | | 0.99 (0.98 – 1.00) | 0.074 |
| Charlson Comorbidity Index | |  |  |
|  | 0 | Reference | ─ |
|  | 1 | 1.24 (0.84 – 1.83) | 0.286 |
|  | 2 | 0.92 (0.62 – 1.35) | 0.655 |
|  | 3 | 1.10 (0.65 – 1.88) | 0.713 |
|  | ≥4 | 0.97 (0.57 – 1.64) | 0.901 |
| Mesh placement | |  |  |
|  | Preperitoneal | Reference | ─ |
|  | Onlay | 3.39 (1.76 – 6.53) | <0.001 |
|  | Retromuscular | 1.63 (0.81 – 3.26) | 0.170 |
|  | IPOM+ | 1.33 (0.68 – 2.61) | 0.400 |
| Defect width >6–10 cm, n = 744 | |  |  |
| Female sex | | 0.98 (0.59 – 1.63) | 0.943 |
| Age, per year | | 0.98 (0.96 – 1.00) | 0.092 |
| Charlson Comorbidity Index | |  |  |
|  | 0 | Reference | ─ |
|  | 1 | 1.30 (0.64 – 2.64) | 0.470 |
|  | 2 | 1.06 (0.50 – 2.26) | 0.874 |
|  | 3 | 2.64 (1.14 – 6.09) | 0.023 |
|  | ≥4 | 1.71 (0.76 – 3.85) | 0.195 |
| Mesh placement | |  |  |
|  | Preperitoneal | Reference | ─ |
|  | Onlay | 5.89 (0.80 – 43.08) | 0.081 |
|  | Retromuscular | 0.98 (0.13 – 7.53) | 0.986 |
|  | IPOM+ | 1.24 (0.16 – 9.71) | 0.838 |

Footnotes: Subgroup analyses for defect widths ≤2 cm, >2–6 cm, and >6–10 cm. HR: hazard ratio; CI: confidence intervals; n: number of patients.

**Table S3** Subgroup analyses for surgical approach

|  | | HR (95% CI) | P value |
| --- | --- | --- | --- |
| Open surgery, n = 3,414 | |  |  |
| Female sex | | 0.90 (0.71 – 1.14) | 0.377 |
| Age, per year | | 0.99 (0.98 – 1.00) | 0.074 |
| Defect width, per cm | | 1.11 (1.06 – 1.16) | <0.001 |
| Charlson Comorbidity Index | |  |  |
|  | 0 | Reference | ─ |
|  | 1 | 1.08 (0.76 – 1.53) | 0.661 |
|  | 2 | 1.15 (0.82 – 1.61) | 0.407 |
|  | 3 | 1.77 (1.16 – 2.72) | 0.008 |
|  | ≥4 | 0.99 (0.61 – 1.59) | 0.964 |
| Mesh placement | |  |  |
|  | Preperitoneal | Reference | ─ |
|  | Onlay | 2.73 (1.52 – 4.91) | <0.001 |
|  | Retromuscular | 1.07 (0.56 – 2.05) | 0.837 |
|  | IPOM+ | 1.37 (0.66 – 2.86) | 0.399 |
| Laparoscopic surgery, n = 1,682 | |  |  |
| Female sex | | 0.92 (0.59 – 1.42) | 0.702 |
| Age, per year | | 0.98 (0.97 – 1.00) | 0.076 |
| Defect width, per cm | | 0.96 (0.86 – 1.08) | 0.487 |
| Charlson Comorbidity Index | |  |  |
|  | 0 | Reference | ─ |
|  | 1 | 1.39 (0.75 – 2.57) | 0.300 |
|  | 2 | 1.45 (0.81 – 2.59) | 0.217 |
|  | 3 | 1.03 (0.39 – 2.72) | 0.955 |
|  | ≥4 | 1.73 (0.80 – 3.73) | 0.162 |
| Mesh placement | |  |  |
|  | Preperitoneal | Reference | ─ |
|  | Retromuscular | 0.67 (0.09 – 5.27) | 0.706 |
|  | IPOM+ | 1.35 (0.70 – 2.62) | 0.369 |
| Robotic surgery, n = 279 | |  |  |
| Female sex | | 0.52 (0.18 – 1.49) | 0.226 |
| Age, per year | | 1.01 (0.97 – 1.06) | 0.603 |
| Defect width, per cm | | 1.02 (0.75 – 1.37) | 0.914 |
| Charlson Comorbidity Index | |  |  |
|  | 0 | Reference | ─ |
|  | 1 | 1.56 (0.47 – 5.17) | 0.465 |
|  | 2 | 0.24 (0.03 – 2.07) | 0.195 |
|  | 3 | 1.04 (0.20 – 5.48) | 0.966 |
|  | ≥4 | 1.48 (0.17 – 12.67) | 0.719 |
| Mesh placement | |  |  |
|  | Preperitoneal | Reference | ─ |
|  | Retromuscular | 1.90 (0.52 – 6.98) | 0.333 |
|  | IPOM+ | 1.01 (0.09 – 10.83) | 0.991 |

Footnotes: Subgroup analyses for surgical approach: open, laparoscopic, and robotic surgery. Onlay mesh placement is only used in open surgery and was therefore not included in the subgroup analyses for the laparoscopic and robotic approaches. HR: hazard ratio; CI: confidence intervals; n: number of patients.

**Table S4** Subgroup analyses for BMI

|  | | HR (95% CI) | P value |
| --- | --- | --- | --- |
| BMI ≤30, n = 2,160 | |  |  |
| Female sex | | 0.73 (0.47 – 1.13) | 0.154 |
| Age, per year | | 0.99 (0.98 – 1.01) | 0.519 |
| Defect width, per cm | | 0.89 (0.78 – 1.01) | 0.073 |
| Charlson Comorbidity Index | |  |  |
|  | 0 | Reference | ─ |
|  | 1 | 1.11 (0.55 – 2.24) | 0.765 |
|  | 2 | 1.65 (0.94 – 2.92) | 0.083 |
|  | 3 | 1.67 (0.78 – 3.54) | 0.186 |
|  | ≥4 | 0.84 (0.32 – 2.24) | 0.733 |
| Mesh placement | |  |  |
|  | Preperitoneal | Reference | ─ |
|  | Onlay | 4.33 (1.54 – 12.15) | 0.005 |
|  | Retromuscular | 3.78 (1.23 – 11.65) | 0.021 |
|  | IPOM+ | 3.60 (1.22 – 10.66) | 0.020 |
| BMI >30, n = 1,075 | |  |  |
| Female sex | | 1.20 (0.66 – 2.17) | 0.547 |
| Age, per year | | 0.99 (0.96 – 1.01) | 0.298 |
| Defect width, per cm | | 1.09 (0.95 – 1.25) | 0.207 |
| Charlson Comorbidity Index | |  |  |
|  | 0 | Reference | ─ |
|  | 1 | 1.02 (0.43 – 2.41) | 0.958 |
|  | 2 | 0.77 (0.31 – 1.88) | 0.562 |
|  | 3 | 1.43 (0.51 – 3.98) | 0.493 |
|  | ≥4 | 1.84 (0.74 – 4.56) | 0.191 |
| Mesh placement | |  |  |
|  | Preperitoneal | Reference | ─ |
|  | Onlay | 2.81 (0.82 – 9.56) | 0.099 |
|  | Retromuscular | 2.15 (0.59 – 7.84) | 0.245 |
|  | IPOM+ | 1.55 (0.43 – 5.60) | 0.507 |

Footnotes: Subgroup analyses for BMI ≤30 and >30. HR: hazard ratio; CI: confidence intervals; n: number of patients.

**Table S5** Subgroup analyses for midline and non-midline incisional hernias

|  | | HR (95% CI) | P value |
| --- | --- | --- | --- |
| Midline incisional hernias, n = 2,198 | |  |  |
| Female sex | | 0.99 (0.72 – 1.35) | 0.936 |
| Age, per year | | 0.99 (0.98 – 1.00) | 0.083 |
| Defect width, per cm | | 1.07 (1.01 – 1.13) | 0.032 |
| Charlson Comorbidity Index | |  |  |
|  | 0 | Reference | ─ |
|  | 1 | 0.69 (0.45 – 1.07) | 0.097 |
|  | 2 | 0.82 (0.51 – 1.33) | 0.419 |
|  | 3 | 1.59 (0.92 – 2.74) | 0.095 |
|  | ≥4 | 0.99 (0.55 – 1.79) | 0.972 |
| Mesh placement | |  |  |
|  | Preperitoneal | Reference | ─ |
|  | Onlay | 2.92 (1.18– 7.23) | 0.021 |
|  | Retromuscular | 0.99 (0.38 – 2.60) | 0.988 |
|  | IPOM+ | 1.28 (0.50 – 3.28) | 0.614 |
| Non-midline incisional hernias, n = 3,177 | |  |  |
| Female sex | | 0.83 (0.63 – 1.09) | 0.181 |
| Age, per year | | 0.99 (0.98 – 1.00) | 0.144 |
| Defect width, per cm | | 1.09 (1.03 – 1.16) | 0.005 |
| Charlson Comorbidity Index | |  |  |
|  | 0 | Reference | ─ |
|  | 1 | 0.98 (0.65 – 1.47) | 0.915 |
|  | 2 | 1.10 (0.70 – 1.73) | 0.681 |
|  | 3 | 1.01 (0.54 – 1.90) | 0.971 |
|  | ≥4 | 0.87 (0.47 – 1.62) | 0.670 |
| Mesh placement | |  |  |
|  | Preperitoneal | Reference | ─ |
|  | Onlay | 2.42 (1.51 – 3.89) | <0.001 |
|  | Retromuscular | 1.64 (0.92 – 2.93) | 0.094 |
|  | IPOM+ | 1.16 (0.70 – 1.93) | 0.563 |

Footnotes: Subgroup analyses for midline and non-midline incisional hernias. HR: hazard ratio; CI: confidence intervals; n: number of patients.

**Table S6** Variation of the main analysis with sublay as the reference

|  | | HR (95% CI) | P value |
| --- | --- | --- | --- |
| Mesh placement | |  |  |
|  | Sublay | Reference | ─ |
|  | Onlay | 2.30 (1.78 – 2.99) | <0.001 |
|  | IPOM+ | 1.05 (0.79 – 1.41) | 0.725 |

Footnotes: Variation of the main analysis with sublay as the reference. Adjusted for sex, age, defect width, and Charlson Comorbidity Index. Sublay: retromuscular and preperitoneal mesh placement combined; HR: hazard ratio; IPOM+: intraperitoneal mesh placement with defect closure.
